# Soft Adaptive Mechanical Metamaterials

**DOI:** 10.3389/frobt.2021.673478

**Published:** 2021-05-03

**Authors:** Romik Khajehtourian, Dennis M. Kochmann

**Affiliations:** Mechanics and Materials Lab, Department of Mechanical and Process Engineering, ETH Zürich, Zürich, Switzerland

**Keywords:** multistability, metamaterial, auxeticity, reconfigurable structures, phase transformation, transition wave

## Abstract

Soft materials are inherently flexible and make suitable candidates for soft robots intended for specific tasks that would otherwise not be achievable (e.g., smart grips capable of picking up objects without prior knowledge of their stiffness). Moreover, soft robots exploit the mechanics of their fundamental building blocks and aim to provide targeted functionality without the use of electronics or wiring. Despite recent progress, locomotion in soft robotics applications has remained a relatively young field with open challenges yet to overcome. Justly, harnessing structural instabilities and utilizing bistable actuators have gained importance as a solution. This report focuses on substrate-free reconfigurable structures composed of multistable unit cells with a nonconvex strain energy potential, which can exhibit structural transitions and produce strongly nonlinear transition waves. The energy released during the transition, if sufficient, balances the dissipation and kinetic energy of the system and forms a wave front that travels through the structure to effect its permanent or reversible reconfiguration. We exploit a triangular unit cell’s design space and provide general guidelines for unit cell selection. Using a continuum description, we predict and map the resulting structure’s behavior for various geometric and material properties. The structural motion created by these strongly nonlinear metamaterials has potential applications in propulsion in soft robotics, morphing surfaces, reconfigurable devices, mechanical logic, and controlled energy absorption.

## 1 Introduction

Soft-bodied animals and actuating plants, which have been perfected through natural evolution, offer abundant bioinspired designs to perform targeted functions (Rus and Tolley, 2015). Soft robotics and programmable materials have taken inspiration from such natural systems, and they have achieved various complex functions and motions through compliant soft composites and mechanical metamaterials with embedded physical intelligence (Saunders et al., 2010; Hines et al., 2017). While the flexibility of soft materials increases their complexity and makes the design of soft robots challenging, it also offers thrilling opportunities to exploit these additional degrees of freedom to devise robotic materials with interactive functionalities.

Over the past decade, various soft robots have been developed, capable of performing different complex types of motion (e.g., quadrupedal locomotion (Shepherd et al., 2011), rolling (Lin et al., 2011), undulation (Onal and Rus, 2013), and jumping (Bartlett et al., 2015)) to mimic caterpillars (Lin et al., 2011), earthworms (Joey et al., 2019), octopuses (Laschi, 2017), snakes (Onal and Rus, 2013), and fishes (Marchese et al., 2014), among others. Usually, the actuators and locomotion mechanisms used in these robots (e.g., dielectric elastomers (Henke et al., 2017), shape memory actuators (Seok et al., 2012), and soft fluidic actuators (Onal and Rus, 2013), internal combustion mechanisms (Bartlett et al., 2015), and motor tendons (Umedachi et al., 2016)) mandate a complex internal architecture as a result of a multistep fabrication and complicated assembly process, in addition to limitations introduced by the weight of control accessories (Marchese et al., 2014).

As a remedy, approaches that allow us to use additive manufacturing techniques and bypass a detailed assembly process have gained attention. Among these approaches is the utilization of elastic instabilities embedded in the robot’s components, which can be triggered by applying elastic deformation or through exposure to environmental stimuli such as pH, moisture, temperature (Kotikian et al., 2019), and light (Zhao et al., 2019). Studies on various *grounded* bi- and multistable metamaterials (Nadkarni et al., 2014; Nadkarni et al., 2016a; Nadkarni et al., 2016b; Raney et al., 2016) and actuators (Chen and Shea, 2018) have shown great potential and resulted in successful realizations of soft robots with embedded bistable mechanisms (Chen et al., 2018).

Multistable metamaterials and actuators are characterized by having more than one stable equilibrium configuration and the possibility to switch between them when a stimulus is applied, without significant changes in their dimensions. While the building blocks of grounded multistable systems are attached to a rigid substrate or support, the fundamental building block of *substrate-free* (ungrounded) multistable metamaterials is also a bi- or multistable unit cell (Rafsanjani and Pasini, 2016), which is, however, attached only to its neighboring cells and not to a ground. As a consequence, unlike their grounded counterparts, substrate-free multistable metamaterials are free to change their shape and dimensions and to move freely through space —additional features, which make them dynamically richer to study, while providing a wide range of opportunities for applications (Jin et al., 2020; Khajehtourian and Kochmann, 2020; Khajehtourian and Kochmann, 2021).

Multistable structures are formed by tessellating periodically or in spatially graded fashion (un)grounded multistable unit cells. When those are initially at rest in a high-energy state (i.e., energetically higher than the minimum-energy equilibrium state of the system), then they support sequential changes in unit cell states, which effects a reconfiguration of the overall structure. First observed for grounded systems, transition fronts form and propagate through the structure in one (Nadkarni et al., 2014) or two dimensions (Frazier and Kochmann, 2017) (1D and 2D, respectively). Similarly, for substrate-free dissipative bistable structures (with dissipation stemming from, e.g., the intrinsic losses of polymer base materials), transition fronts can act as a topological soliton (Jin et al., 2020), which gradually transforms the structure from unswitched to switched upon propagation. In conventional solitons, an equilibrium between nonlinearity and dispersion leads to a stable wave (Hussein and Khajehtourian, 2018; Khajehtourian and Hussein, 2019). By contrast, the topological solitons in dissipative ungrounded multistable metamaterials emerge from the balance between dissipation and kinetic energy, on the one hand, and the energy released when switching unit cells from high-energy (open) stable state to their low-energy (closed) one, on the other hand. This energetic balance results in a constant-velocity propagating front, which separates two regions of different strain states (Khajehtourian and Kochmann, 2020). Such substrate-free multistable metamaterials can be conceived from a wide range of bi- and multistable unit cells and different soft structural mechanisms in 1D, 2D, and 3D (Khajehtourian and Kochmann, 2021).

**FIGURE 1:**
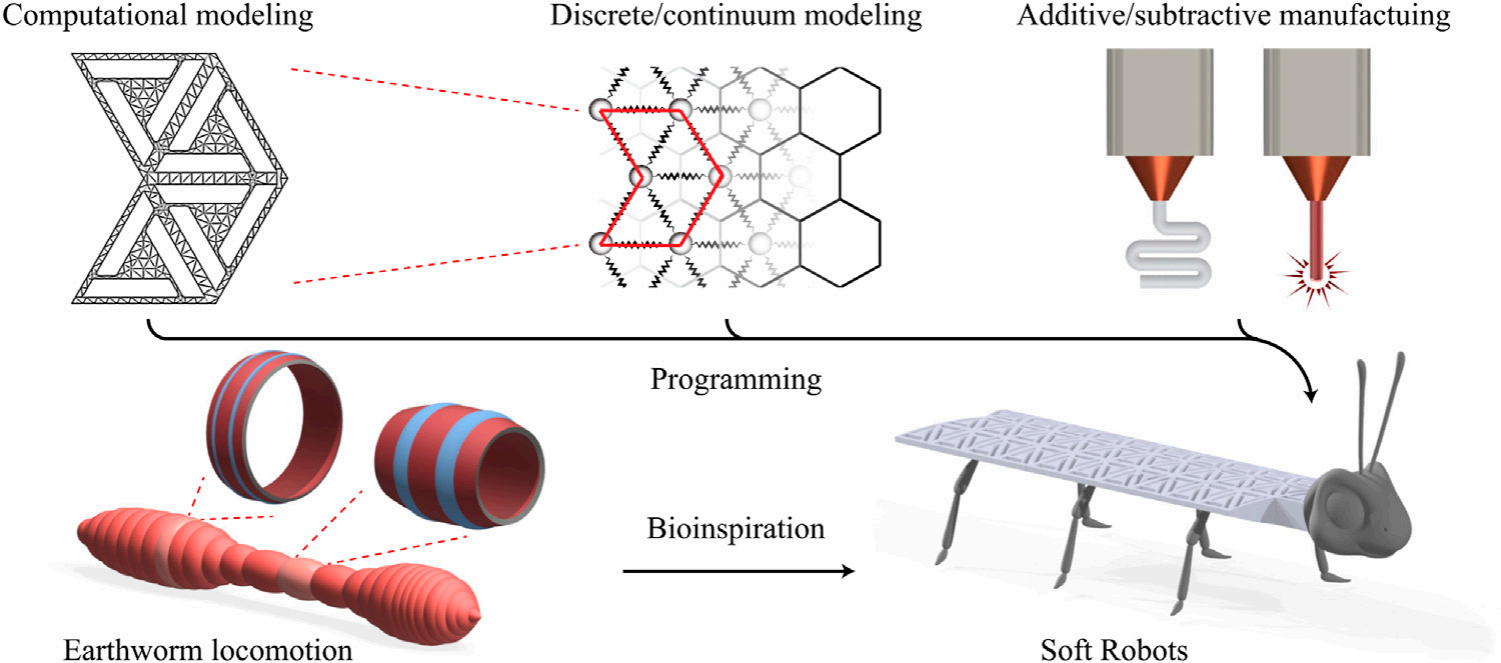
Bioinspired soft robots *via* adaptive mechanical metamaterials. Bistable unit cell architectures in conjunction with a powerful discrete/continuum model provide a rich medium for programming and manufacturing soft robotic materials.

Here, we summarize recent research on substrate-free reconfigurable metamaterials from the soft robotic application’s perspective. Understanding these metamaterials’ mechanics and dynamics provides useful tools for harnessing, designing, and deploying such mechanisms in soft robots (Figure 1). They support structural reconfiguration and, upon a careful design, exhibit complex motions, for example, anchor pulling or serpentine, which is of importance for soft robotic applications. In the remainder of this report, we describe the multistable unit cell of interest and provide parametric studies using finite element analysis (FEA). We briefly describe our continuum model for these multistable structures and finally present new designs before concluding with an outlook.

## 2 Soft Adaptive Mechanical Metamaterials

There are several mechanisms that rise to soft substrate-free multistable metamaterials: unit cells with rotating components (Rafsanjani and Pasini, 2016), buckling beams (Yang and Ma, 2020), and shells (Tan et al., 2020). By tessellating any such unit cell on a lattice, one constructs a reconfigurable structure. Manufacturing metamaterials based on buckling beams or shells typically requires the individual fabrication of unit cells and their subsequent assembly, while those metamaterials based on designs as in Rafsanjani and Pasini (2016) are free of assembly and are made by merely introducing cut patterns into a sheet, resembling kirigami.

**FIGURE 2:**
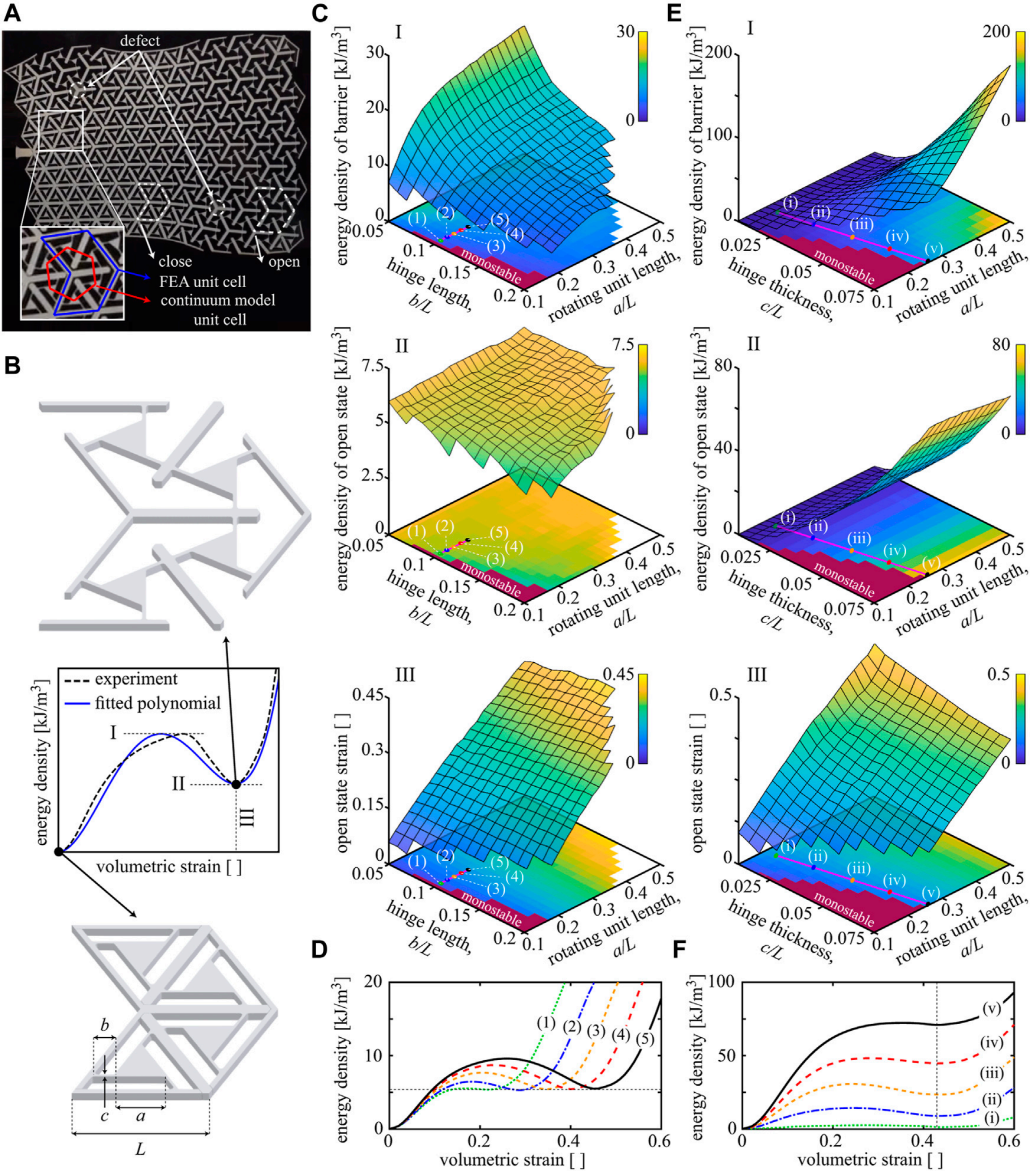
Architectural parameters control the bistability. **(A)** A 2D defected multistable structure with a gradual transformation of unit cells from their open configuration to the closed one. This structure was produced by introducing cuts into a polymer sheet (Jin et al., 2020). **(B)** When a single unit cell under volumetric straining is bistable, the strain energy density exhibits two stable minima associated with the closed ($\varepsilon_{0}$) and open ($\varepsilon_{s}$) configurations. The energy density is approximated by a fourth-order polynomial characterized by the energy density barrier (I), the energy density of the open state (II), and the open-state strain (III). The unit cell’s architecture is characterized by the unit cell length *L*, rotating unit length *a*, hinge length *b*, and thickness *c*. **(C)** Design maps of strain energy characteristics for varying hinge and rotating unit lengths while keeping the hinge thickness constant at c=0.025L. **(D)** Double-well strain energy densities corresponding to the colored markers in the design maps of Figure 2C. **(E)** Design maps of bistability characteristics for varying hinge thicknesses while keeping the hinge length constant at b=0.1L. **(F)** Double-well strain energy densities corresponding to the markers in the design maps of Figure 2E.

As a representative fundamental building block, we consider a triangular unit cell with embedded rotating units that have two stable equilibria, the manufactured (closed) configuration and a volumetrically strained (open) configuration (see Figure 2A). When strained, this unit cell experiences a volumetric expansion, which arises from the combined effects of rotating and displacing close-to-rigid components, overall accommodating the soft reconfiguration and bistability. This behavior results in a double-welled potential strain energy with each well corresponding to one of the two stable equilibria. While the as-manufactured state’s energy is zero, the unit cell upon expansion stores strain energy in its rotating components’ thin hinges, resulting in a non-zero higher energy (Figure 2B). Where needed, we can alter individual unit cells by forcing them to remain in one of their stable configurations, for example, by adding spacers that fill the gaps between the components of an open unit cell, preventing it from collapsing into its closed state (referred to as a defect). We characterize the unit cell architecture by their unstretched length *L*, rotating unit length *a*, hinge length *b*, and thickness *c*. The thickness of the sheet is defined by *d*. The resulting periodic structures are classically made by introducing periodic cuts into soft sheets such as acetal homopolymer or natural latex rubber (Rafsanjani and Pasini, 2016; Jin et al., 2020). Alternatively, they can be additively manufactured through multi-material printing of rigid components of a stiff material and connecting the latter through soft hinges.

Reconfiguration and transition front propagation can be modeled through the homogenization approach introduced by Khajehtourian and Kochmann (2021). Rather than modeling each and every structural member in the unit cells, this approach efficiently approximates the discrete structure by a continuum, whose efficiency is of importance for simulating large systems and especially for design optimization. This continuum model requires as inputs the unit cell’s nonconvex potential in addition to its dissipation potential and the base material’s elastic stiffness. In practice, we extract the nonconvex strain energy density of a unit cell as a function of an applied volumetric strain by conducting FEA on individual unit cells under periodic boundary conditions, whose response is validated by experiments (Jin et al., 2020). The resulting approximate continuum model is governed by linear momentum balance, that is,

$$\rho \ddot{u}=\nabla \cdot \sigma \;\text{with}\;\sigma =\sigma^{\text{visc}.}+\sigma^{\text{inter}.},$$ (1)

which is to be solved for the continuous displacement field u=u(x,t) defined at time *t* at every point x∈Ω within a body Ω having uniform mass density ρ. Both viscous and elastic interaction stresses stem from unit cell interactions. Let the neighboring cells of any given cell in the periodic array be located in the (unit) directions ${\hat{R}}_{j}$, and let $\hat{N}$ denote the set of all such direction vectors ${\hat{R}}_{j}$ (only counting one for each pair of symmetric neighbors). For example, for the triangular lattice shown in Figure 2A, the three nearest neighbors are located at ${\hat{R}}_{1}=\left(1,0\right)$ and ${\hat{R}}_{2,3}=\left(\pm 1/2,\sqrt{3}/2\right)$. (Note that the FEA unit cell area is twice the area of the unit cell in the continuum model (see Figure 2A), which requires proper scaling of the energy density.) In such a setup, following the derivations of Khajehtourian and Kochmann (2021), the elastic interaction stress tensor contribution can be derived from an effective strain energy density Ψ in general as

$$\sigma^{\text{inter}.}=\frac{\partial \text{Ψ}}{\partial \varepsilon }\left(\varepsilon \right)\;\text{with}\;\text{Ψ}\left(\varepsilon \right)=\sum_{j\in \hat{N}}\psi_{j}\left({\hat{R}}_{j}\cdot \varepsilon {\hat{R}}_{j}\right),$$ (2)

where ε=sym(∇u) is the (total) infinitesimal strain tensor and $\psi_{j}$ represents the interaction energy with the *j*th neighbor. Since the bistability of the chosen unit cell is associated with volumetric expansion, we decompose the total strain into $\varepsilon_{\text{vol}}=\text{tr}\varepsilon /2$ and $\varepsilon_{\text{dev}}=\varepsilon -\varepsilon_{\text{vol}}I$ to further decompose the effective strain energy density into volumetric and deviatoric contributions as follows:

$$\text{Ψ}\left(\varepsilon \right)=\sum_{j\in \hat{N}}\left[\chi_{j}\left({\hat{R}}_{j}\cdot \varepsilon_{\text{vol}}{\hat{R}}_{j}\right)+{ϒ}_{j}\left({\hat{R}}_{j}\cdot \varepsilon_{\text{dev}}{\hat{R}}_{j}\right)\right].$$ (3)

It is convenient to approximate the volumetric two-well potential (for all interactions in a centrosymmetric lattice) by the quartic polynomial $\chi_{j}\left(\varepsilon \right)=\chi \left(\varepsilon \right)=c_{2}\varepsilon^{2}+c_{3}\varepsilon^{3}+c_{4}\varepsilon^{4}$ with constants $c_{i}\in \mathbb{R}$ defined for strain ε between neighboring cells, and we further choose ${ϒ}_{j}\left(\varepsilon \right)=ϒ\left(\varepsilon \right)=\mu \varepsilon^{2}/2$ with shear stiffness μ > 0. Analogously, the viscous interaction stresses derived from the effective dissipation potential density (with strain rate $\dot{\varepsilon }=\;\text{d}\varepsilon /\text{d}t$) are

$$\sigma^{\text{visc}.}=\frac{\partial \text{Φ}}{\partial \dot{\varepsilon }}\left(\dot{\varepsilon }\right)\;\text{with}\;\text{Φ}\left(\dot{\varepsilon }\right)=\frac{\beta }{2}\sum_{j\in \hat{N}}{\left({\hat{R}}_{j}\cdot \dot{\varepsilon }{\hat{R}}_{j}\right)}^{2},$$ (4)

with viscosity β>0. Overall, the resulting linear momentum balance for the lattice based on a triangular unit cell becomes

$$\rho \ddot{u}=\nabla \cdot \left[\frac{3}{8}\beta \left(2\dot{\varepsilon }+I\text{tr}\dot{\varepsilon }\right)+\frac{3}{2}\chi^{\prime }\left(\varepsilon_{\text{vol}}\right)I+\frac{3}{4}\mu \varepsilon_{\text{dev}}\right].$$ (5)

For a 1D substrate-free bistable chain, the potential difference between the two stable states at $\varepsilon_{0}=0$ and $\varepsilon_{s}$, that is, $\text{Δ}\chi =\chi \left(\varepsilon_{s}\right)-\chi \left(\varepsilon_{0}\right)$ is related to the velocity of the transition front through a scaling law (Khajehtourian and Kochmann, 2020) (which was shown to hold for various choices of ρ and β). Since the energy density barrier height $\chi \left(\varepsilon_{b}\right)$ is important in initiating a transition wave, we obtain constants $c_{i}$ by setting $\chi \left(\varepsilon_{0}\right)=\varepsilon_{0}=0$ and fitting χ(ε) to match $\chi \left(\varepsilon_{b}\right)$, $\chi \left(\varepsilon_{s}\right)$, and $\varepsilon_{s}$ from experiments or FEA (cf. the dashed lines and bistable curves of Figure 2B).

## 3 Results and Discussion

In what follows, we present results and discuss the key features of adaptive bistable metamaterials toward soft robotic applications. We exploit the triangular unit cell’s architecture when loaded under uniaxial tension to show how varying the unit cell geometry affects the energy landscape. We further demonstrate the effects of unit cell distribution and of structural and material parameters on the structure’s overall motion and reconfigurability. Finally, we show a few selected examples highlighting how complex motion can be achieved, suitable for a wide range of applications, among them soft robotics.

We extract the double-well energy density *χ* for the triangular unit cells of Figure 2B by performing FEA, using the commercial package ABAQUS. The base material is acetal; material properties and sheet *d* are adopted from Jin et al. (2020). We use an elastoplastic constitutive model calibrated with uniaxial tensile test data (Jin et al., 2020) and discretize each unit cell *via* finite strain quadratic plane stress elements. We consider contacts through nonlinear penalty constraints to avoid localized penetration of unit cell components, especially around the hinges which undergo large deformations; the unit cell is loaded by strain-driven periodic boundary conditions. We use a dynamic implicit solver to determine the quasistatic response. For a unit cell with *L* = 0.027 m, we vary the dimensions of *a*, *b*, and *c* and compute the resulting strain energy density *χ*.

The strain energy density as a function of volumetric strain allows us to control the unit cell’s (multi-)stability. A unit cell is bistable, if the strain energy density has a second local minimum at some non-zero strain as shown in Figure 2B. FEA enables us to build a design map for $\chi \left(\varepsilon_{\text{vol}}\right)$ by simulating different unit cell geometries. When varying hinge and rotating unit lengths (while fixing the hinge thickness at c=0.025L), the energy density level of the open state remains almost constant (see Figure 2C). By contrast, when changing hinge thickness and rotating unit length (at fixed hinge length b=0.1L), the open-state energy density increases with hinge thickness (see Figure 2E). Hence, the main parameter responsible for the energy level of the open state is the hinge thickness. Overall, when increasing the rotating unit length, the open-state strain increases linearly. This value is essential for tuning the stroke length of soft robots made out of such unit cells (Chen and Shea, 2018). The energy density barrier, however, depends on all dimensional parameters. Cases in which $\chi \left(\varepsilon_{b}\right)-\chi \left(\varepsilon_{s}\right)\leq 0$ are monostable, whereas small energy differences values $\chi \left(\varepsilon_{b}\right)-\chi \left(\varepsilon_{s}\right)>0$ may be preferable as they require little energy input for triggering the transition from open to closed cells. The shown design maps are further beneficial for recognizing comparable energy densities of open states or their strains, especially when designing graded structures based on spatially varying unit cells to manipulate the transition front propagation. Examples of smoothly varying energy densities are shown in Figures 2D,F. As a further metric of practical interest, we observe that the stiffness upon straining past the open configuration is approximately independent of the parameter choices.

Moving on to structures and based on the continuum approximation of *Soft Adaptive Mechanical Metamaterials*, we discretize all structures *via* finite elements (using 2D constant-strain triangular elements) and employ variational constitutive updates with a backward Euler finite difference approximation of strain rates to solve Eq. 5 by a Newmark-β implicit scheme using Newton–Raphson iteration at each time step. Defects are implemented *via* stiff bar elements superimposed on top of the structural elements. In compliance with experimental measurements, we assume negligible body forces and mass density.

**FIGURE 3:**
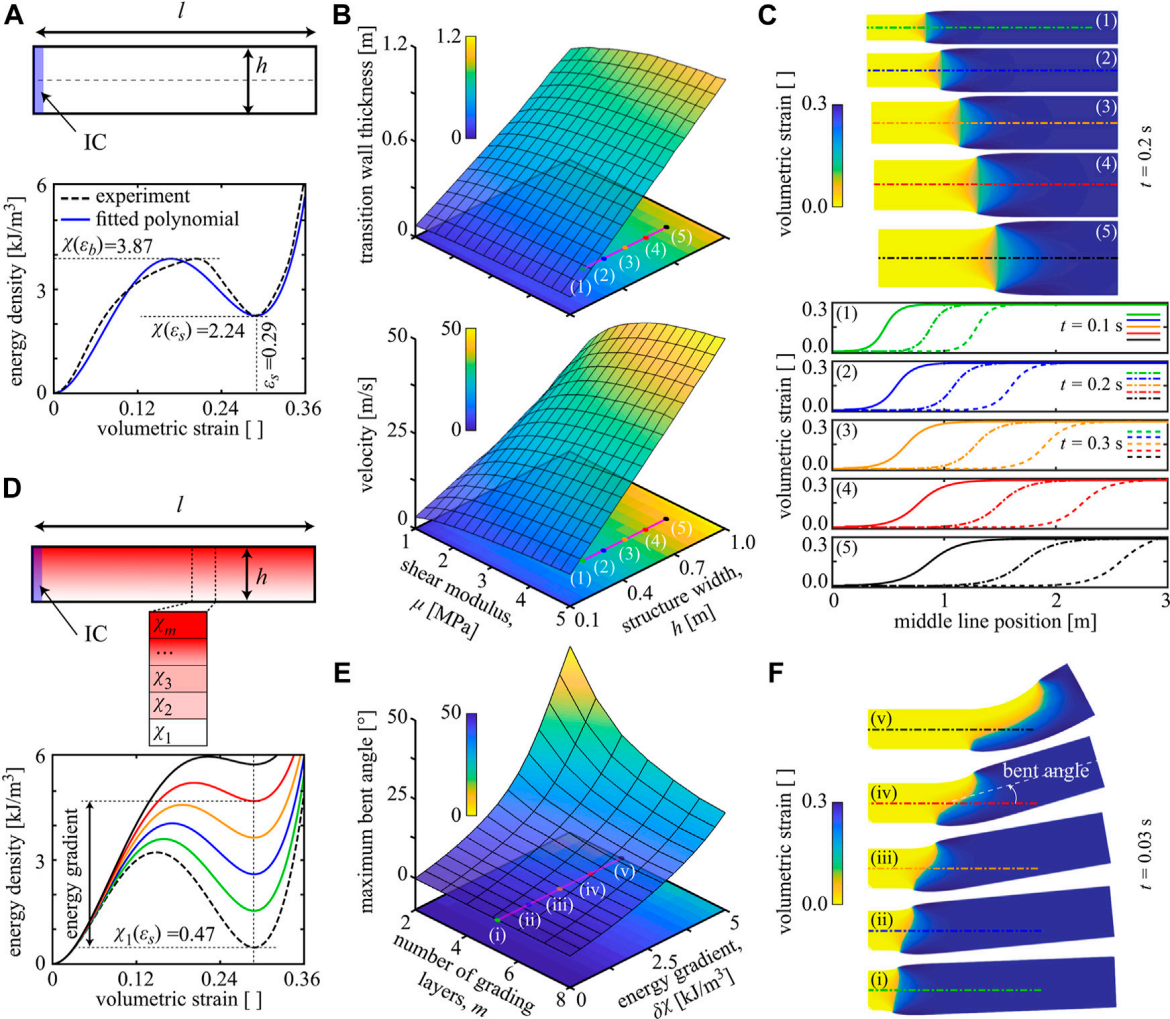
Bistability and structural dimensions control the reconfiguration. **(A)** A structure with *l* = 3 m and varying *h* is initially in its open state, except for the highlighted area with the initial condition (IC) of being in the closed state. This initiation causes a transition front propagating from left to right. Double-well energy density extracted from Jin et al. (2020) and its quartic polynomial fit. **(B)** The velocity and thickness of the transition wave for varying structural width *h* and shear modulus μ values. **(C)** Snapshots of the simulated transitioning structure and the wave profile at their center line, corresponding to the marked points in Figure 3B, at the three instances of *t* = 0.1 s (solid line), *t* = 0.2 s (dash-dotted line), and *t* = 0.3 s (dash-dotted line). **(D)** A rectangular structure with *l* = 8*h* is made from unit cells whose geometry (dimensions) changes vertically across the structure; unit cells at the bottom have the dashed energy density curve, while cells at the top have the solid black energy curve (at fixed μ=1 MPa). **(E)** Maximum bent angle of the graded structure in **(D)** as a function of the energy differential $\delta \chi =\chi_{m}\left(\varepsilon_{s}\right)-\chi_{1}\left(\varepsilon_{s}\right)$ and the number of grading layers *m*. **(F)** The graded structure’s motion corresponding to the marked values of Figure 3E at *t* = 0.03 s (for β/Δt=0.2 MPa).

Let us explore the effects of material properties, structural dimensions, and unit cell distribution on the structural response by highlighting a few selected examples. Starting from the open state of a structure, we simulate an impact by setting the unit cells in a region marked by “initial conditions” (IC) into their closed configuration (see Figure 3A). We here use the bistable energy density $\chi \left(\varepsilon_{\text{vol}}\right)=\left(585000\varepsilon_{\text{vol}}^{2}-3677630\varepsilon_{\text{vol}}^{3}+6041910\varepsilon_{\text{vol}}^{4}\right)$, obtained from fitting to experiments Jin et al. (2020). As shown in Figure 3B, the transition front becomes wider and propagates faster close to linearly, when we increase the width of the structure (normal to the front). Increasing the shear modulus increases both the transition front width (measured at the center line) and the front velocity. Figure 3C shows examples of transition fronts and their profiles at the center line for different parameters. These effects highlight that the dynamics of substrate-free metamaterials depend not only on the base material and architecture but also on the structural dimensions.

Intriguing structural reconfiguration can be achieved when going beyond periodic tessellations and instead spatially varying the unit cell architecture to achieve targeted motion. In Figure 3D, we consider a rectangular structure in which we gradually change the unit cell design across the width by a total of *m* equal thickness horizontal layers (each having uniform unit cells but the unit cell design varying from layer to layer with the energies shown in Figure 3D). We assign the lowest layer’s unit cells to an architecture with a low-energy level $\chi_{1}\left(\varepsilon_{s}\right)$ in its open state and gradually increase this value toward the *m*th (top) layer of the structure with the second energy well at $\chi_{m}\left(\varepsilon_{s}\right)$. The structure is initially in its open configuration. Upon initiation from the left, the propagation speed increases with the local energy release so that the transition front moves fastest in the top layer and slowest at the bottom. As a consequence, the structure bends (Figure 3F). Figure 3E illustrates how the maximum bent angle (defined by the normals on both ends of the structure) varies with the number *m* of layers and the energy gradient $\delta \chi =\chi_{m}\left(\varepsilon_{s}\right)-\chi_{1}\left(\varepsilon_{s}\right)$ for a constant thickness of the structure. The bent angle grows with increasing energy gradient, contrary to increasing the number of layers. Example structures with different bent angles are shown in Figure 3F. This design type introduces transverse motion, which can be a basis for programming locomotion modes.

**FIGURE 4:**
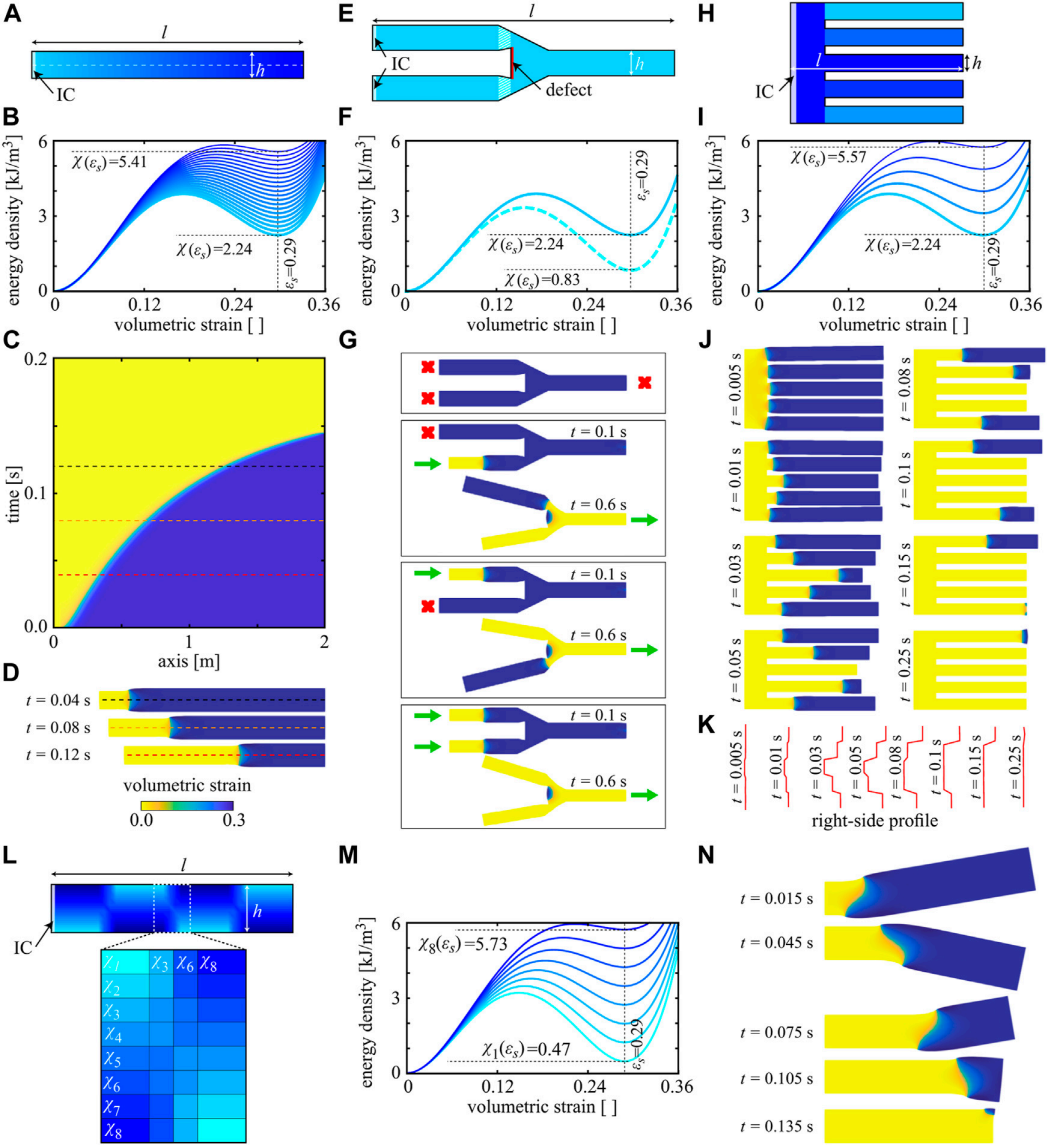
Transition effects on reconfigurability. **(A)** A spatially graded slab (unit cells vary in the horizontal direction) with dimensions *l* = 10*h* having linearly varying values of the open-state strain energy **(B)** along its length. **(C)** When impacted, the structure reveals a smoothly accelerating transition in the *x*-*t*-diagram of the center line, with three snapshots of the reconfiguration shown in **(D)**. **(E)** A tuning fork-like structure (*l* = 12*h*) made from two distinct underlying architectures (color code matches with curves in **(F)**) and a rigid defect demonstrating OR logic **(G)**. **(H)** A comb-like structure (*l* = 10*h*) made from multiple distinct underlying unit cells (color code matches with curves in **(I)**), which enables careful control of the delay between signals propagating in figures **(J)** and temporal programming of the signal arrival at the right end of the structure **(K)**. **(L)** A rectangular structure (*l* = 8*h*) with alternating unit cell gradients along the length and smooth gradients along the width (color code matching the energy densities in **(M)**; eight layers along the width and four layers between each section along the length). **(N)** When impacted, the structure undergoes serpentine locomotion. (All results for β/Δt=0.2 MPa and μ=1 MPa.)

Highlighting the combined effects of material properties, unit cell design, and structural geometry, we proceed to explore potential applications of substrate-free multistable metamaterials in 2D, particularly for soft robotics. In Figure 4A, we consider a slender rectangular structure with smoothly varying unit cell dimensions (similar to Figure 2F), resulting in a structure with a linearly graded design along its main axis. The associated, spatially varying, double-well energy potential along the structure is shown in Figure 4B. Increasing $\chi \left(\varepsilon_{s}\right)$ increases the velocity of the transition front, as shown analytically in Khajehtourian and Kochmann (2020). Hence, grading the architecture of the structure (or geometry width, or even the base material) allows us to manipulate the transition speed in a smooth fashion; as an example, Figure 4C illustrates a smoothly accelerating front suitable for energy propeller and absorber applications (Supplementary Video S1).

Following the same principle, that is, manipulating the transition speed *via* altering the underlying unit cell, one can design mechanical logic gates. We define the open configuration as a logical state “1” and the closed state as a logical state “0,” and we design a soft mechanical OR gate. In the bifurcated structure of Figure 4E, we plant a rigid defect in the forked area and alter the unit cell near the defect such that more energy is required to overcome the energy barrier $\chi \left(\varepsilon_{b}\right)-\chi \left(\varepsilon_{s}\right)$ (Figure 4F) at the defect. The structure is initially in its open configuration. When initiated from either of the input branches on the left-hand side of the structure, the released energy from the transition and the front velocity are sufficiently high to overcome the altered unit cells’ energy barrier. However, when the transition front passes that area, it slows down considerably and cannot overcome the other branch’s altered energy barrier (Supplementary Video S1). Therefore, the front stops from propagating in the other input branch, while it continues to the output branch (see Figure 4G). Of course, initiating the impact in both input branches provides sufficient energy to activate the output branch as well (thus resulting in a typical OR behavior). Designs that require a large amount of energy to overcome the barrier can hence beneficially act as defects.

Variations in the transition speed further enable the preprogramming of time-dependent reconfiguration sequences. To demonstrate this concept, we consider the comb-like structure of Figure 4H, whose branches are populated with different unit cell designs according to Figure 4I. The structure is initially in its open configuration. Upon impaction on the structure’s left, a transition front forms and propagates at different velocities in each branch. This allows us to engineer the wave profile reaching the right end and the exact time delays between individual signals reaching the right end through the five branches (Supplementary Video S1). Snapshots of a propagating transition front and the evolving right-edge profiles are shown in Figures 4J,K.

Finally, complex sequences of structural motion can be achieved by consulting the design map of Figure 3E for the design of 2D and 3D structures. In Figure 4L, we consider a rectangular structure divided into four sections along the length *l* with alternating unit cell gradients across the width (graded with eight layers of changing unit cell designs). All unit cells have similar open-state strains but varying energy barriers (Figure 4M). We further smooth the unit cell changes between each section with a four-layer gradient along the length of the structure. When initiated from the left, the resulting motion in Figure 4N resembles serpentine locomotion, which serves as a representative example of designing soft robots that perform preplanned, time-dependent maneuvers (Supplementary Video S1).

## 4 Conclusion and Future Directions

Exploiting instability has been a major theme across engineering mechanics for about a decade. At the structural level, the existence of multiple stable configurations has been exploited in, among others, mechanical logic, and reconfigurable and deployable structures. We here discussed the design of substrate-free multistable metamaterials, whose nonlinear, time-dependent response was shown to be intimately tied to the underlying unit cell architecture and the chosen base material. Through numerical simulations, we predicted the response of a number of representative examples to rationalize the complex configurations that arise in the structures’ post-buckling regime after different loading and boundary conditions. The chosen numerical approach allows us to extract any unit cell’s energy landscape and to characterize its features which are linked to the structural response in terms of, for example, stroke length, execution speed, and the amount of energy required for actuation. For faster transition, longer strokes and energy release are preferable, while the energy barrier should be minimized without removing the multistability entirely.

We leveraged the recently introduced continuum description of Khajehtourian and Kochmann (2021), which serves as a reduced-order surrogate for simulating the effective response of large arrays of (periodic or, approximately, spatially graded) unit cells and makes for efficient simulations that bypass fully resolved discrete structural calculations. Using the continuum model, we showed examples demonstrating the potential of the chosen multistable structural design, including structures that smoothly accelerate or decelerate mechanical waves, bifurcated structures with implanted defects which act as mechanical logic gates, complex geometries capable of preprogrammed reconfiguration, and 2D graded structures exhibiting serpentine motion.

Of course, the presented soft architectures have limitations. Reconfigurable metamaterials based on stiff base materials provide low density and friction, which are beneficial for soft robotic applications. However, their performance life is limited by the occurrence of plastic deformation (and eventually failure) of hinges under cyclic loading, which may be a limiting factor for repeated use in soft robots. Softer base materials such as natural rubber may overcome this limitation, yet they lack dimensional stability in variable environmental conditions. We used a quartic polynomial to model the bistability while recognizing that a more complex double-well representation might perform closer to experimental results. Our simulations neglected friction between the structure and the surface on which it is moving; while those factors could restrict the reconfiguration and motion (Jin et al., 2020), they also provide fruitful grounds for further preplanning and optimizing target motion (e.g., by designing spatially variable friction coefficients or optimizing ground adhesion by structural designs). We also assumed (supported by experimental observation) that in our overdamped scenario, the mass density is negligible; if a structure’s mass is significant, the transition phenomenon persists but linear waves (such as elastic precursor shocks (Khajehtourian and Kochmann, 2020)) may warrant consideration.

The fundamental building blocks and concepts for reconfigurable metamaterials discussed here provide a basis for the design of soft robots moving, for example, *via* the anchor pulling or serpentine locomotion. For designs based on substrate-free multistable unit cells, this study is intended to serve as a guide. The particular unit cells and parameter variations considered here are only the tip of the iceberg. A careful exploration of the wide design space of unit cell architectures in 1D, 2D, and 3D, of spatially varying designs and base materials, of symmetry-breaking defects and free surfaces, and of complex shaping (e.g., tessellating 2D unit cells of the type described here onto 3D shell networks) offers a rich playground for soft robotic applications with as-designed time-dependent behavior. A few examples of 2D and 3D unit cells for bistable architectures are summarized in Khajehtourian and Kochmann (2021). By utilizing active base materials such as, for example, shape-memory polymers, the same principles can be extended to structures that respond to temperature or light without requiring a mechanical stimulus. Multi-physics couplings such as the inclusion of (electro-) magnetic elements or long-range couplings by connecting remote unit cells are further relatively unexplored terrains. Topology optimization and data-driven approaches could aid in the fast and efficient performance optimization as well as in the inverse design. Of course, the theoretical design of such mechanisms and architectures must be closely tied to experimental reality and the constraints arising from fabrication techniques. Yet, current trends in increasing resolution, scalability, and material selection and combination in advanced manufacturing techniques give hope to an exciting research direction. Since the response of the shown architected materials is essentially scale-free, their design principles apply over a wide range of scales, from transformable meter-scale architectures to tunable nanostructures (the latter might display material level size effects, which, however, can also be beneficially exploited).

## Data Availability

The raw data supporting the conclusions of this article will be made available by the authors, without undue reservation.
